# Orientation anisotropy of quantitative MRI relaxation parameters in ordered tissue

**DOI:** 10.1038/s41598-017-10053-2

**Published:** 2017-08-29

**Authors:** Nina Hänninen, Jari Rautiainen, Lassi Rieppo, Simo Saarakkala, Mikko Johannes Nissi

**Affiliations:** 1 Department of Applied Physics, University of Eastern Finland, POB 1627, FI-70211 Kuopio, Finland; 2 Research Unit of Medical Imaging, Physics and Technology, University of Oulu, POB 5000, FI-90014 Oulu, Finland; 3 Medical Research Center Oulu, Oulu University Hospital and University of Oulu, Oulu, Finland; 4 Department of Diagnostic Radiology, Oulu University Hospital, Oulu, Finland

## Abstract

In highly organized tissues, such as cartilage, tendons and white matter, several quantitative MRI parameters exhibit dependence on the orientation of the tissue constituents with respect to the main imaging magnetic field (B_0_). In this study, we investigated the dependence of multiple relaxation parameters on the orientation of articular cartilage specimens in the B_0_. Bovine patellar cartilage-bone samples (*n* = 4) were investigated *ex vivo* at 9.4 Tesla at seven different orientations, and the MRI results were compared with polarized light microscopy findings on specimen structure. Dependences of T_2_ and continuous wave (CW)-T_1ρ_ relaxation times on cartilage orientation were confirmed. T_2_ (and T_2_*) had the highest sensitivity to orientation, followed by T_RAFF2_ and adiabatic T_2ρ_. The highest dependence was seen in the highly organized deep cartilage and the smallest in the least organized transitional layer. Increasing spin-lock amplitude decreased the orientation dependence of CW-T_1ρ_. T_1_ was found practically orientation-independent and was closely followed by adiabatic T_1ρ_. The results suggest that T_1_ and adiabatic T_1ρ_ should be preferred for orientation-independent quantitative assessment of organized tissues such as articular cartilage. On the other hand, based on the literature, parameters with higher orientation anisotropy appear to be more sensitive to degenerative changes in cartilage.

## Introduction

Orientation anisotropy describes the dependence of a measured parameter on the orientation of the measured object or material. In the case of magnetic resonance imaging (MRI), nuclear magnetic properties of tissue depend not only on its chemical characteristics and macroscopic structure, but also on the physical organization of macromolecules^1^. Highly organized tissues, such as skeletal muscle^2^, tendon^3, 4^, white matter^5, 6^ and cartilage^7^, exhibit different properties when MRI measurements are conducted at different tissue depths or at different physical orientations with respect to the primary magnetic field^8–10^. Multiple underlying physical or chemical properties of tissue may act as a source for the anisotropy in different MRI parameters^1^. While the anisotropy of relaxation affects various tissues, as listed above, in this study we focus on the orientation dependence of relaxation parameters in articular cartilage. Specifically the deep cartilage, which has a uniform and highly organized structure, may serve as a generic model for ordered tissue.

At microscopic scale, articular cartilage consists of a network of collagen fibres and proteoglycan molecules, which together comprise the extracellular matrix^11^. Orientation anisotropy of MRI parameters in cartilage arises from the structure of the collagenous meshwork in which the fibres have a specific depth-wise orientation and organization^7, 12, 13^. Non-calcified cartilage can be divided into three structural zones on the basis of the depth-wise change of collagen fibre orientation^14–16^. Starting from the articular surface, the first zone is the superficial or tangential zone (SZ) with fibres running mostly parallel to the surface. Below this, the fibres have a more random orientation in the transitional (intermediate) zone (TZ), and finally in the radial (deep) zone (RZ), the orientation has turned perpendicular to the surface. In the deep zone, the structure of cartilage is the most organized, resulting also in the highest anisotropy of its properties^11, 13, 17^.

Articular cartilage functions as a shock-absorbing tissue and enables low-friction during joint movement^18^. Osteoarthritis (OA) is a disease which affects a growing number of people, and results in progressive degeneration of articular cartilage and the surrounding tissues in joints^19^. There is no cure for advanced OA, but the progression of the disease may be slowed down by dietary choices and weight management^20^. Thus, diagnosing OA at the earliest possible stage is essential in preventing the progression and minimizing the symptoms^20^. While numerous quantitative MRI methods have been proposed and established for early diagnosis of OA^21–24^, a significant issue persists that the results of many of those (particularly T_2_ relaxation time) are susceptible to orientation anisotropy^1, 25, 26^.

In almost every joint of the human body, articular cartilage is naturally at variable orientations, causing different regions of cartilage to be scanned at different angles with respect to the MRI scanner’s magnetic field; thus yielding potentially variable results for the different regions of otherwise similar cartilage. In practice, the measurement geometry due to joint shapes cannot be controlled and, therefore, orientation-independent MRI methods or detailed understanding of the dependence would be necessary to avoid possible false diagnoses and to allow realistic analysis of the structure and state of the health of cartilage.

The sensitivity of different quantitative MRI (qMRI) parameters to relaxation anisotropy varies. T_1_ has been shown not to be sensitive to orientation^7^, whereas T_2_ and continuous wave (CW)-T_1ρ_ have been shown to have excessive sensitivity to orientation^27–29^. For CW-T_1ρ_, reduction of orientation sensitivity in cartilage has been reported for increasing spin-lock powers^27, 30^.

Orientation dependence of T_2_ relaxation time in articular cartilage has been ascribed to residual dipolar interactions (RDI) of water protons due to their restricted spatial arrangement within the collagen fibres^17, 26^. In addition to water proton intramolecular dipolar interactions, intermolecular dipolar couplings between water and biopolymer protons have also been suggested as a source of T_2_ anisotropy^10, 31^. Dipolar interaction between two nuclei is directly proportional to the factor (3 cos^2^ θ − 1), where θ is the angle between the direction of the magnetic field and a vector joining the nuclei^8^. Dipolar interaction is one of the predominant relaxation mechanisms and is generally noticed as a signal reduction except at θ angles near or at so called “magic angle” of 54.74° where (3 cos^2^ θ − 1) = 0 and the dipolar interaction vanishes^8^. Thus, qMRI relaxation time parameters affected by dipolar interaction reach maximum values at this specific angle and show a (3 cos^2^ θ − 1)-dependence on the sample orientation^7^.

Practical value of qMRI parameters depends on their potential and accuracy for diagnosis of diseases, such as OA. Recently, T_1ρ_ and T_2_^32^, and rotating frame relaxation (RFR) methods including adiabatic T_1ρ_, adiabatic T_2ρ_ and T_RAFF2_^24, 33^, have been reported to be sensitive for cartilage degeneration^24, 33^. Orientation sensitivities of these parameters have been investigated in a preliminary study; adiabatic T_2ρ_ and T_RAFF2_ were observed to have a similar orientation sensitivity as T_2_, whereas adiabatic T_1ρ_ was shown to be less sensitive to orientation than T_2_, both in *ex vivo* and *in vivo* measurements^34, 35^.

The aim of the experimental part of this study was to investigate the orientation dependence of several quantitative MRI parameters (T_1,_ T_2,_ T_2_*, CW-T_1ρ_, adiabatic T_1ρ_, adiabatic T_2ρ_ and T_RAFF2_) and evaluate the findings against the collagen fibre orientation and anisotropy, measured by the gold standard reference technique, i.e., quantitative polarized light microscopy (qPLM). As a secondary aim, the usefulness of the aforementioned parameters for OA diagnostics, as described and reported in the literature, was evaluated against the orientation sensitivity determined in the experimental part.

## Results

**Figure 1:**
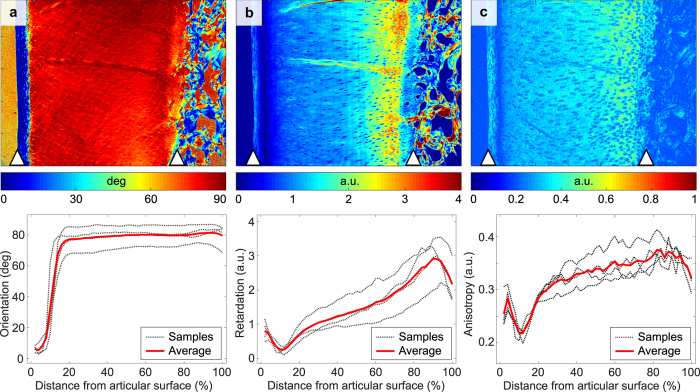
PLM results presented as a map for one representative sample and profiles of all the specimens: (**a**) orientation; (**b**) retardation; (**c**) anisotropy. Articular surface and cartilage-bone interface are marked on the maps with arrowheads. Profiles were calculated separately for each sample along cartilage depth and averaged point-by-point for mean profile.

qPLM revealed the typical tri-laminar collagen orientation in all the samples, starting with fibres oriented along the cartilage surface and arching towards radial orientation at the cartilage-bone interface (Fig. 1a). Similar to qPLM anisotropy, optical retardation was lowest in the transitional zone and increased towards the deep tissue, indicating increasing anisotropy (Fig. 1b,c).

**Figure 2:**
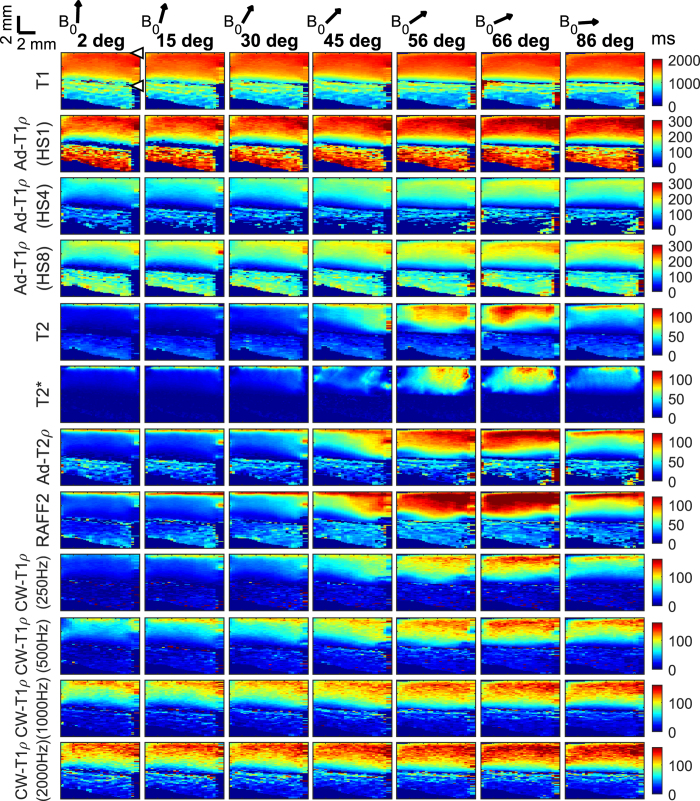
Relaxation parameter maps for one representative sample at different angles with respect to B_0_ (arrows above). Orientation anisotropy is clearly seen for T_2_, T_2_*, Ad-T_2ρ_ and T_RAFF2_. Articular surface and cartilage-bone interface are marked with arrowheads.

**Figure 3:**
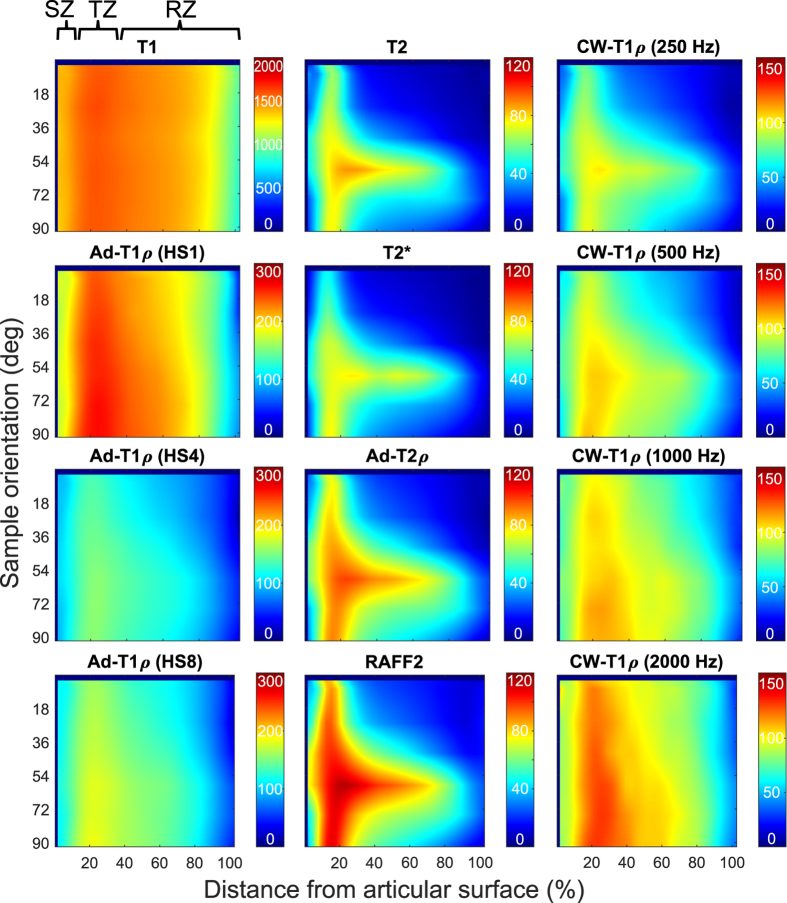
Interpolated profile maps for relaxation parameters (one representative sample). Articular surface is on the left, bone interface towards right, with the profiles at different orientations stacked on top of each other and then interpolated. SZ, TZ and RZ denote the approximate locations of the histological zones.

Different sensitivities of the parameters to sample orientation with respect to B_0_ were observed (Figs 2 and 3). Orientation dependence was absent or minimal for T_1_, adiabatic T_1ρ_ with HS1 pulse and CW-T_1ρ_ at 2 kHz spin-lock amplitude. On the other hand, using HS4 or HS8 pulses for adiabatic T_1ρ_, or decreasing the spin-lock power of CW-T_1ρ_ increased the orientation dependence. T_2_, T_2_*, adiabatic T_2ρ_ and T_RAFF2_ had the highest sensitivity to orientation, visualized by the largest changes over the orientation, especially in the deep tissue. Identical behaviour was observed with all the samples. The regional minimum, maximum and mean values of the relaxation times over all the measurements reflected the same observations on orientation sensitivities (Table 1).

**Table 1:** Mean and min/max values of the relaxation parameters (in ms) in the different cartilage zones and SNR values through all samples and all orientations. Scan times (in minutes) are for one sample per single orientation.

	SZ	TZ	RZ	SNR	Scan time
T_1_	1425 (1266/1544)	1534 (1406/1657)	1367 (1287/1489)	34.8	5:50
Adiabatic T_1ρ_ (HS1)	193 (150/232)	236 (200/278)	189 (167/224)	70.8	4:11
Adiabatic T_1ρ_ (HS4)	126 (94/174)	155 (129/191)	113 (80/145)	62.4	4:11
Adiabatic T_1ρ_ (HS8)	129 (102/162)	160 (136/192)	117 (84/151)	63.1	4:11
T_2_	42 (23/71)	60 (38/98)	26 (6/68)	32.5	4:11
T_2_*	38 (20/73)	52 (30/79)	22 (5/55)	37.0	2:14
Adiabatic T_2ρ_	58 (33/91)	75 (48/115)	35 (10/80)	48.0	3:30
RAFF2	69 (40/110)	85 (57/135)	40 (14/96)	54.8	6:51
CW-T_1ρ_ (250 Hz)	60 (29/97)	77 (37/129)	39 (14/85)	41.1	3:23
CW-T_1ρ_ (500 Hz)	73 (51/106)	91 (63/125)	53 (27/90)	44.9	3:23
CW-T_1ρ_ (1000 Hz)	83 (62/112)	103 (83/125)	72 (47/97)	48.6	3:23
CW-T_1ρ_ (2000 Hz)	95 (69/118)	115 (92/138)	88 (70/108)	51.3	3:23

**Figure 4:**
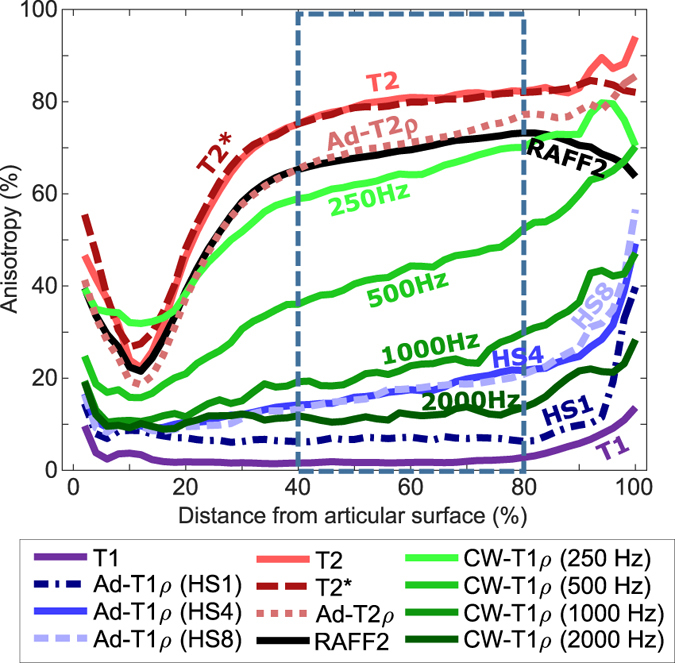
Depth-wise MR anisotropy profiles for relaxation parameters (average of four samples). Smallest anisotropy is noted at the location of the transitional zone, while the maximum anisotropy is observed in the deep zone. Boxed area shows the depth used for calculating deep cartilage average values.

Analysis of the depth-wise MR anisotropy, as determined using the Michelson contrast parameter, revealed distinctive differences between the relaxation times (Fig. 4). For those parameters that demonstrated sensitivity, the anisotropy changed as a function of depth: the minimum was observed at the transitional zone, higher relaxation anisotropy at the surface and the maximum in the radial zone, closely resembling the qPLM anisotropy of the collagen network (Fig. 1c). Relaxation anisotropy of the MRI parameters was assessed as a bulk value in the radial zone with the expected most uniform collagen architecture and the highest qPLM anisotropy. T_1_ relaxation anisotropy in this region had the lowest correlation with qPLM anisotropy (*r* = 0.03) and retardation (*r* = −0.10), followed by adiabatic T_1ρ_ with HS1 pulse (*r* = 0.09/−0.10), whereas the anisotropies of T_2_ and T_2_* relaxation times had the highest correlations (*r* = 0.87/−0.88 and *r* = 0.87/−0.86, respectively) (Table 2).

**Table 2:** Average anisotropy of the relaxation parameters in deep cartilage (average of four samples and range (min/max)) and the coefficients of correlation for the depth-wise MRI anisotropy with depth-wise PLM anisotropy and PLM retardation. Averages for correlation coefficients have been calculated using Fisher’s z transform. The parameters are ordered in the table based on their anisotropy.

	Average MRI Anisotropy	MRI Anisotropy vs. PLM Anisotropy	MRI Anisotropy vs. PLM Retardation
T_2_	80.0 (77.8/85.3)	0.87 (0.82/0.90)	−0.88 (−0.95/−0.77)
T_2_*	79.4 (75.3/86.9)	0.87 (0.71/0.93)	−0.86 (−0.94/−0.68)
Adiabatic T_2ρ_	71.3 (66.0/81.4)	0.89 (0.86/0.91)	−0.87 (−0.93/−0.82)
RAFF2	69.6 (64.2/76.5)	0.90 (0.81/0.96)	−0.86 (−0.92/−0.77)
CW-T_1ρ_ (250 Hz)	64.6 (53.9/77.0)	0.84 (0.71/0.92)	−0.77 (−0.89/−0.54)
CW-T_1ρ_ (500 Hz)	43.6 (30.2/54.5)	0.80 (0.63/0.88)	−0.72 (−0.81/−0.60)
CW-T_1ρ_ (1000 Hz)	22.6 (16.9/32.5)	0.69 (0.52/0.83)	−0.58 (−0.65/−0.48)
Adiabatic T_1ρ_ (HS4)	17.6 (14.0/21.8)	0.55 (0.24/0.88)	−0.46 (−0.66/−0.18)
Adiabatic T_1ρ_ (HS8)	17.3 (11.7/24.9)	0.52 (0.13/0.80)	−0.46 (−0.58/−0.28)
CW-T_1ρ_ (2000 Hz)	12.0 (9.4/15.7)	0.34 (−0.12/0.66)	−0.35 (−0.59/0.13)
Adiabatic T_1ρ_ (HS1)	6.8 (6.0/8.4)	0.09 (−0.19/0.44)	−0.10 (−0.25/0.08)
T_1_	1.9 (1.1/2.5)	0.03 (−0.06/0.14)	−0.10 (−0.17/−0.06)

**Figure 5:**
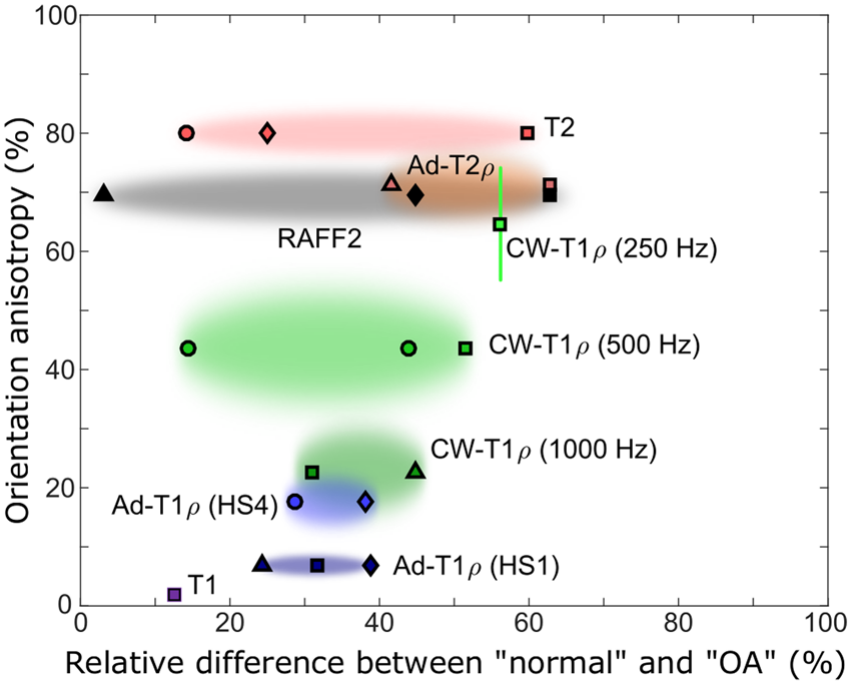
Average anisotropy of relaxation parameters in deep cartilage versus relative difference of relaxation parameters between “normal” and “OA” in different models as reported in literature (o = human *in vivo* ^45, 60, 61^, □ = human *ex vivo* ^24^, ∆ = *in vivo* animal OA model with imaging done for samples^33^, ◊ = *ex vivo* animal model^55^). Shaded area represents deviation in anisotropy and range of relative difference values. For T_2_*, Adiabatic T_1ρ_ with HS8 pulses, and CW-T_1ρ_ 2000 Hz no reference values were found. The optimal parameter would lie in the lower right corner.

Assessing the anisotropies of the MRI parameters in the radial zone enabled sorting the parameters by their respective sensitivities to the specimen orientation in the main field (Fig. 5, Table 2). Based on values reported in the literature, relative differences between “normal” or “intact” and variably degenerated or degraded (“OA”) animal and human tissue were evaluated and plotted together with the estimated orientation anisotropies (Fig. 5). Broadly, parameters with higher sensitivity to orientation anisotropy also demonstrated largest relative differences between intact and degenerated articular cartilage (Fig. 5).

## Discussion

Orientation dependence of several quantitative traditional and rotating frame MRI parameters in ordered tissue was investigated in this study. The findings on the MR anisotropy of several quantitative relaxation time parameters were compared to measurements of structural anisotropy of cartilage tissue by qPLM. Furthermore, literature-reported relative differences for the investigated MR parameters between intact and degenerated tissue were evaluated against the assessed orientation sensitivities.

In this study, we selected articular cartilage as a model tissue that has both highly anisotropic and also relatively isotropic regions^7^. The current findings confirmed earlier reports on the orientation sensitivity of T_1_ and T_2_ relaxation times^7, 17, 36^, and the reduction of the orientation sensitivity with increasing spin-lock power for CW-T_1ρ_^27, 30^. Orientation dependence was observed also for other relaxation parameters, and was the most significant for T_2_, T_2_*, adiabatic T_2ρ_ and T_RAFF2_.

As previously demonstrated in multiple studies, T_2_ relaxation time along with T_2_* was the most sensitive to orientation of the sample, a phenomenon ascribed to the residual dipolar interaction in cartilage and other similar highly organized tissues^17, 27, 37, 38^. T_2_ relaxation time is sensitive to dipolar interactions and, thus, this was an expected finding. T_2_*, on the other hand, might be sensitive also to field inhomogeneity contributions from anisotropic susceptibility of the collagen fibres in the cartilage^39, 40^, since it carries not only T_2_ but also the inhomogeneity related component. However, very similar relaxation anisotropy to T_2_ was noted for T_2_*.

The orientation dependencies of adiabatic T_1ρ_, adiabatic T_2ρ_ and T_RAFF2_ have been briefly investigated previously for articular cartilage^34^. The present results demonstrated the orientation dependence of T_RAFF2_ and adiabatic T_2ρ_ to be very similar to that of T_2_ or T_2_*, as was noted in the preliminary findings^34^. Furthermore, an increase in the dependence for adiabatic T_1ρ_ was observed with increasing pulse stretching factor *n* ^41^. Three different pulse modulations using hyperbolic secant function with stretching factors of 1, 4 and 8 were tested. To allow comparison, the RMS powers and bandwidths of the pulse modulations were kept the same (approximately equal to 906 Hz CW-pulse power). As the pulse is stretched further, the shape approaches that of a CW-pulse and the time the magnetization spends in the vicinity of the xy-plane is increased, i.e. the contributions from off-resonance T_1ρ_ relaxation are reduced and more dipolar relaxation is allowed, explaining the observed change in anisotropy^41–44^. However, even with the HS8 pulse modulation, the relaxation was nearly orientation-independent.

Native (non-contrast enhanced) T_1_ relaxation time and adiabatic T_1ρ_ with HS1 pulses were found to be essentially independent of the tissue orientation. Adiabatic T_1ρ_ and T_2ρ_ have been shown to be sensitive to changes in cartilage even *in vivo*, and adiabatic T_1ρ_ seems promising also with clinical MRI equipment^45, 46^. The independence of T_1_ and adiabatic T_1ρ_ parameters on the orientation of the tissue promotes them as suitable parameters to be applied *in vivo* for assessing tissue status. For quantitative MR imaging of articular cartilage, this also implies that the dGEMRIC method^47^, while requiring exogenous contrast agent, should provide orientation-independent assessment. Similar orientation-independence is expected for other T_1_-based contrast-enhanced imaging methods.

Assuming dipolar interaction is the main contributor to spin relaxation in cartilage, both longitudinal T_1_ and transverse T_2_ relaxation depend on molecular fluctuations introducing field perturbations driving the relaxations^7^. Compared with T_1_, T_2_ relaxation has an additional contribution from the secular component of the dipole-dipole interaction (relaxation depending on the spectral density at zero frequency, which does not affect the T_1_ relaxation). This additional component brings about the orientation sensitivity to T_2_ relaxation. In sufficiently organized matter, such as cartilage, this orientation dependent component may not completely vanish by random molecular motion and represents itself as orientation-dependent T_2_ relaxation. Since T_1_ relaxation does not have this component, it is not orientation dependent. This also suggests an explanation for the orientation insensitivity of the adiabatic T_1ρ_ relaxation. With an adiabatic pulse, the magnetization follows the trajectory of the RF field; this results in the magnetization relaxing along the RF field. The RF modulation of the adiabatic HS-pulse is such that the effective field starts along the Z-axis, halfway through the pulse traverses through the transverse plane and ends along the opposite direction of the Z-axis^41, 44^. Thus the relaxation during an adiabatic HS-pulse can be viewed as a combination of longitudinal and transverse relaxations, with the amounts of the respective components dependent on the pulse shape. This is also evidenced by the increased orientation sensitivity of adiabatic T_1ρ_ when the pulse is stretched, making the effective field (and the magnetization) spend more time in the transverse plane and thus experiencing more T_2_-like relaxation.

For all the parameters with clear anisotropy along the cartilage depth, the minimal orientation dependence was detected in the transitional zone, at approximately 12–14% of depth from the articular surface. This is the zone in which the anisotropy of the collagen fibres is also at its minimum. Above this zone, in the superficial cartilage, the anisotropy is higher due to the preferential arrangement of the fibres along the surface. However, collagen fibres in the superficial cartilage are also distributed at multiple orientations along the plane parallel to the surface^48, 49^. Thus, orientation dependent relaxation times depend also on the rotation angle about the axis of the surface normal. In the radial zone, however, the collagen fibres are more uniformly oriented along the axis of the surface normal and relaxation is less affected by the rotation about this axis. This also explains the observed maximum of the qMRI anisotropy in the radial zone. The overall average relaxation times and their ranges in the different zones generally reflect the same observations, although the averages can only be considered indicative and descriptive of the measurements due to the different orientation sensitivities of the parameters.

Since the deep cartilage has the most uniform collagen fibre structure^13, 17^ (see also Fig. 1), it was chosen as a region to represent the overall orientation sensitivity of the relaxation parameters. Orientation anisotropy in the radial zone was calculated for all the parameters as the average value from the depth of 40% to 80%. According to qPLM data, this region is safely within the limits of approximately constant fibre angle, and represents only the deep cartilage without the influence of transitional zone or cartilage-bone interface.

The results of the qPLM orientation and retardation are in accordance with the results of Xia *et al*.^50^ and Rieppo *et al*.^13^ on mature articular cartilage. On average, the difference between orientations of fibres in the superficial and radial zone was approximately 75 degrees, which is less than the theoretical value of 90 degrees. This likely reflects the actual variations in the fibre orientation in the deep zone, as well as the possibility that the primary orientation can differ from the angle exactly perpendicular to the cartilage-bone interface or cartilage surface^51^.

The change in orientation anisotropy for CW-T_1ρ_ followed the previously reported change: increasing spin-lock amplitude reduced the sensitivity^27^, with the conclusion that spin-lock amplitude greater than 500 Hz starts to overpower the RDI at 1.5 T. Here it was found that 500 Hz already reduces the sensitivity to orientation (i.e. to RDI), but does not remove it; the sensitivity is further reduced with 1 kHz and 2 kHz amplitudes (see also Fig. 4). Mlynarik *et al*. tested CW-T_1ρ_ at two different B_0_ field strengths and concluded that dipolar interaction is a major factor in T_1ρ_ relaxation especially at lower field strengths^26^. However, in practice the increasing SAR values of increasing spin-lock amplitude (and often also hardware limitations) prevent the clinical use at high frequencies (typically up to ~500 Hz is clinically applicable)^52^.

The maximum values for the relaxation times (Fig. 3) were found at sample orientations of 59°, 56°, 69° and 72°. Theoretically, the dipolar relaxation vanishes at the magic angle, which is 54.7°^8^. The observed small deviations are probably reflective of the small variations in the actual fibre angles in deep cartilage with respect to the sample itself, as the orientations of the specimens were determined from the scout images based on the orientation of the cartilage surface^15^. Recognizing this possibility, there was no specific attempt to scan the specimens exactly at the magic angle, potentially also allowing missing of the true maximum, which might lie between the measured angles.

As a step forward from standard anatomical imaging, qMRI has a huge potential to provide improved diagnostics and objective, quantitative information of tissue properties at the molecular level. However, in clinical MRI, the measurement geometry often cannot be altered. Thus, orientation independent MRI methods, or understanding of the dependence, is necessary to avoid possible false diagnoses and to allow realistic analysis of the structure and of the state of the tissue being imaged^53^. The correlation (or lack of it) of the anisotropy of the qMRI parameters with the PLM anisotropy effectively indicates the sensitivity of the different parameters to orientation and further to the geometry of the scan setup. To precisely characterize relaxation changes in ordered tissue, more orientations are required for those parameters that correlate with structural anisotropy. From the literature comparison (Fig. 5) it can be seen that the parameters with the best sensitivity in detecting tissue changes related to OA also tend to have the highest sensitivity to orientation anisotropy. This suggests that the methods sensitive to the orientation are thus also sensitive to changes in the orientation, i.e. sensitive to the properties of the collagen fibre network, which is one of the primary components of articular cartilage. Thus, sensitivity to orientation anisotropy may have a role in the sensitivity of the parameters for detecting differences between “intact” or “normal” and “degenerated” tissue. However, the optimal qMRI parameter would exhibit zero sensitivity to orientation while having the maximum sensitivity to tissue degeneration or changes in tissue, i.e., parameters approaching lower right corner in Fig. 5 would be generally preferred. While a number of different MRI parameters were studied, yet more possibilities exist: for example, magnetization transfer experiments have been demonstrated to have minimal or no orientation dependence^54^. On the other hand, sensitivity to orientation changes could provide an alternative means to analyse tissue structure and properties at the molecular level.

## Conclusion

In conclusion, adiabatic T_1ρ_, also reported sensitive to cartilage degeneration^24, 33, 55^, appeared as a promising, minimally orientation-dependent quantitative MRI parameter for the articular cartilage. Additionally, adiabatic pulses provide flexibility with respect to overcoming SAR issues. While limited and somewhat variable results have been reported for native T_1_, it appears as the most promising parameter out of the investigated in terms of SAR and orientation (in)dependence. This finding also suggests that other measurements based on estimating T_1_ relaxation time (such as contrast enhanced methods) would be promising. The practical usefulness of qMRI parameters depends on how well they can be used in diagnosis of diseases, such as OA. Based on the literature findings and the measured relaxation anisotropies, parameters with higher sensitivity to orientation anisotropy generally also demonstrated larger relative differences between intact and degenerated articular cartilage. The best-suited MRI parameter for diagnostics in ordered tissues remains uncertain.

## Methods

### Sample Preparation and MRI Measurements

Cylindrical osteochondral plugs (*n* = 4, diameter = 6 mm) from bovine patella were prepared (bovine knee joints were obtained from a local abattoir; the exact age of the animals is not known, but the typical age at the time of slaughter is approximately 18 months). The samples were immersed in perfluoropolyether (Galden HS 240, Solvay Solexis, Italy) in a custom-built holder, which allowed (manual) rotation of the specimens *i.e*. rotation of cartilage surface normal axis with respect to the main imaging magnetic field (B_0_) from outside the scanner. MRI was performed at 9.4 T using a 19 mm quadrature RF volume transceiver and VnmrJ3.1 Varian/Agilent DirectDrive console. The samples were imaged at room temperature at seven different orientations, spanning 0–90 degrees at approximately 15 degree intervals with respect to B_0_. The orientation was confirmed from scout images and later precisely measured (cartilage surface normal vs. B_0_) from 3-D gradient echo (GRE) data acquired for every orientation. Relaxation time measurements were realized using a global preparation block coupled to a single slice fast spin echo readout (TR = 5 s, ESP = 5.5 ms, ETL = 8 with centric echo ordering, matrix = 256 × 64, FOV = 16 × 16 mm and 1mm slice, yielding a resolution of 62.5 µm along cartilage depth). A single imaging slice was positioned at the centre of the specimen, perpendicular to the axis of specimen rotation, and was rotated with the specimen. The measurements included T_1_ relaxation time with inversion recovery (TI = 0.2, 0.5, 0.8, 1.1, 1.4 and 3 s); T_2_ with spin echo preparation (TE = 10, 20, 40, 80, 100 and 128 ms); CW-T_1ρ_ with four spin-lock amplitudes (γB_1_/2π = 250, 500, 1000 and 2000 Hz)^56^; adiabatic T_1ρ_ with pulses from the hyperbolic secant HS*n* family (HS1, HS4 and HS8 pulses, τ_p_ = 4.5 ms, and γB_1max_/2π = 2.5, 1.2 and 1.04 kHz, respectively, to match the RMS power between the pulse shapes (equal to approximately 906 Hz CW-pulse))^41^; adiabatic T_2ρ_ with HS1 pulse train embedded between adiabatic half passages and T_RAFF2_^57^ (γB_1,max_/2π = 625 Hz, τ_p_ = 9 ms). CW-T_1ρ_ was measured with spin-lock durations of 0, 24, 48, 96 and 192 ms; both adiabatic T_1ρ_ and T_2ρ_ were measured with pulse trains of 0, 4, 8, 12 and 24 pulses using MLEV4 phase cycling, and T_RAFF2_ with trains of 0, 2, 4, 6 and 8 pulses acquired with and without an inversion preparation^55, 56^. Finally, T_2_* relaxation time was measured in the same slice using a multi-echo GRE sequence (TE = 2.5, 7.5, 12.5, 17.5, 22.5, 27.5, 32.5 and 37.5 ms). Scan times together with the overall SNR values for the sequences are given in Table 1. All measurements were repeated for every orientation. After the MRI studies, the samples were fixed in 10% neutral buffered formalin for 48 hours and then decalcified in EDTA for histology processing.

### Histology and quantitative polarized light microscopy (qPLM)

After decalcification, the samples were cut in the same plane that was used in MR imaging utilizing 3-D reconstructions of the MRI data as visual guides. Subsequently, the samples were dehydrated and embedded in paraffin. Unstained histological sections were prepared from the locations of the MRI slices and placed on the standard microscope slides. The sections were digested with hyaluronidase enzyme for 18 hours to remove proteoglycans before quantitative polarized light microscopy (qPLM) measurements (pixel size 2.53 × 2.53 µm). The optical retardation and collagen fibre orientation in the sections were measured according to Rieppo *et al*.^13^ using quantitative Abrio PLM imaging system. Abrio PLM system (CRi, Inc., Woburn, MA, USA) is mounted on a conventional light microscope (Nikon Diaphot TMD, Nikon, Inc., Shinagawa, Tokyo, Japan) and consists of a green bandpass filter, a circular polarizer, a computer-controlled analyser composed of two liquid crystal polarizers and a CCD camera.

### Data Analysis

In MRI analyses, to obtain relaxation time maps, relaxation time constants were fitted in a pixel-wise manner using the respective signal equations: T_RAFF_ was fitted using exponential decay and approach to steady state^57^, CW-T_1ρ_ using 2-parameter exponential with baseline and the rest of the parameters were fitted using 2-parameter monoexponentials with noise floor subtraction before fitting, using in-house developed Matlab (Matlab R2015b, Mathworks, Natick, MA, USA) plugin functions for Aedes (http://aedes.uef.fi) (examples of fit curves and values can be found in the supplementary Figure S1 and Table S1). Depth-wise profiles of 1.75 mm width from the cartilage surface to bone interface were calculated at the centres of the specimens. The profiles were then depth-normalized for comparison and averaging between specimens.

In qPLM analyses, anisotropy of the collagen fibres was calculated by applying an entropy filter of size 5 × 5 pixels (entropyfilt, Matlab) to the orientation angle images obtained from the qPLM. The anisotropy is represented by the inverse of the entropy + 1 to constrain the values to the range [0, 1]. The orientation, retardation and anisotropy profiles were calculated separately for each sample along cartilage depth and averaged point-by-point for mean profile and depth-normalized for comparison. Average values and ranges for the relaxation times over the samples and orientations were calculated in the three structural zones, SZ, TZ and RZ (regions shown in Fig. 3).

Parallelism, or anisotropy of the collagen fibres, can be defined as Michelson contrast^13, 58^:

$${A}_{i}=\frac{{R}_{i}^{max}-{R}_{i}^{min}}{{R}_{i}^{max}+{R}_{i}^{min}},$$

where *min* and *max* are the minimum and maximum measured intensity *R*_*i*_ over the different physical orientations of the specimen. Here, this formalism was used to calculate the depth-wise MR anisotropy profiles for the relaxation parameters using the relaxation rates at different sample orientations as the measured intensities.

To quantitatively analyse relaxation anisotropy of the MRI parameters as a bulk property, an average value for the depth of 40% to 80% was calculated, since the deep cartilage, i.e., radial zone has relatively constant fibre orientation with the highest anisotropy (Fig. 4, ROI region shown with dashed line).

Correlations between the MR anisotropy profiles and PLM measurements were calculated per sample and then averaged using Fisher’s z transform^59^. Matlab was used for all calculations and analyses.

To assess the orientation dependence versus relative sensitivity of the parameters, the average anisotropies of the MR parameters were plotted against relative difference of the same parameters between early OA and advanced OA, or intact and degenerated tissue, taken from multiple previous studies reporting different OA cases or OA models^24, 33, 45, 55, 60, 61^.

### Data Availability

All of the raw data, documentation and analysis codes of the study are available for download at Zenodo (doi:10.5281/zenodo.519752).

## Electronic supplementary material

Supplementary Information 
