# Supplementary material for: Orientation anisotropy of quantitative MRI relaxation parameters in ordered tissue
Source: Sci Rep. 2017 Aug 29;7:9606. doi: 10.1038/s41598-017-10053-2 (PMC5574987; doi:10.1038/s41598-017-10053-2)
Supplement: Supplementary file 1 — Supplementary Information [file 41598_2017_10053_MOESM1_ESM.pdf]

Supplementary Information for:

Orientation anisotropy of quantitative MRI relaxation parameters in ordered tissue

Hänninen Nina[1,2], Rautiainen Jari[1], Rieppo Lassi[2,3], Saarakkala Simo[2,3,4] and Nissi Mikko Johannes[1]*

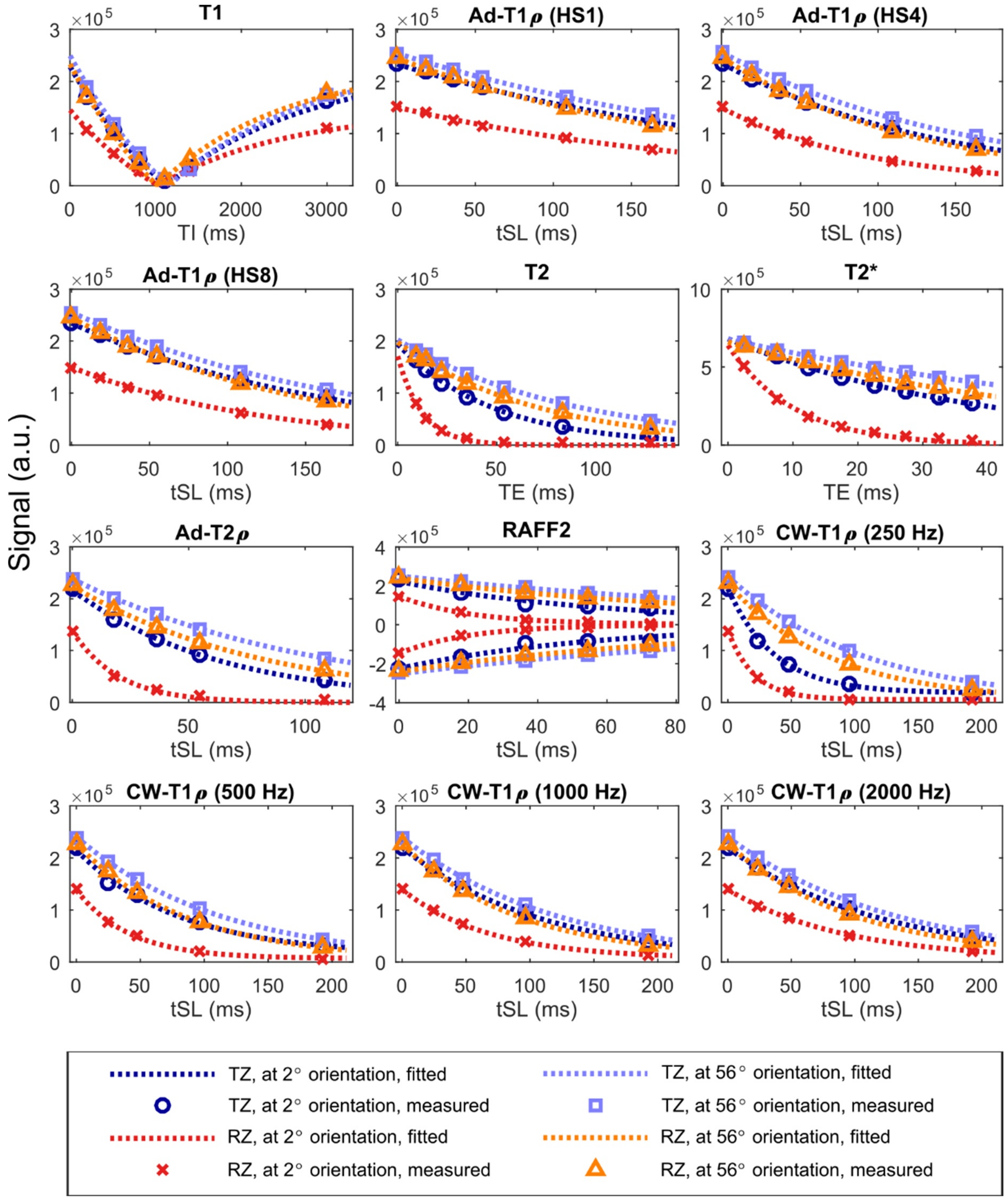

Supplementary Figure S1. Examples of fitting curves for the different relaxation parameters in transitional zone (TZ) and radial zone (RZ) for one representative sample at two different orientations (2° and 56°). Dashed lines represent the fit curves and symbols the measured data points. Corresponding fit values are given in supplementary table S1 below.

Supplementary Table S1. Examples of the fit values for the different relaxation parameters, at two different orientations (2 and 56 degrees) and in the transitional zone (TZ) and radial zone (RZ) of cartilage. Values are for one representative sample. Fitting error is the second norm distance between the fit and the measured signal, relative to the maximum of the raw signal.

	TZ							
	At 2° orientation				At 56° orientation			
	Fit	S_0	†BL / S_{SS}	error (%)	Fit	S_0	†BL / S_{SS}	error (%)
T_1	1625	228162		5.1 %	1661.7	249384.0		4.7 %
Adiabatic $T_{1\rho}$ (HS1)	256	233424		0.5 %	269.3	253916.4		0.5 %
Adiabatic $T_{1\rho}$ (HS4)	144	233970		0.4 %	162.1	254531.6		0.9 %
Adiabatic $T_{1\rho}$ (HS8)	171	234177		0.3 %	185.6	255048.9		0.3 %
T_2	48	193839		4.8 %	89.6	202009.2		1.9 %
T_2*	40	680206		1.1 %	73.2	677128.3		0.6 %
Adiabatic $T_{2\rho}$	64	216568		2.1 %	105.4	238459.1		0.4 %
RAFF2	59	224650	5948	18.8 %	120.8	251760.6	14578	3.3 %
CW-$T_{1\rho}$ (250 Hz)	37	201108	18339	1.7 %	106.6	243336.7	~0	0.5 %
CW-$T_{1\rho}$ (500 Hz)	80	201300	13934	5.6 %	112.5	240636.6	~0	0.7 %
CW-$T_{1\rho}$ (1000 Hz)	111	218511	1437	1.2 %	121.5	239732.0	~0	0.5 %
CW-$T_{1\rho}$ (2000 Hz)	120	213527	5727	1.0 %	132.9	239808.6	~0	0.6 %

	RZ							
	At 2° orientation				At 56° orientation			
	Fit	S_0	†BL / S_{SS}	error (%)	Fit	S_0	†BL / S_{SS}	error (%)
T_1	1489	145123		4.9 %	1472	233275		5.2 %
Adiabatic $T_{1\rho}$ (HS1)	213	150573		0.8 %	218	244089		0.1 %
Adiabatic $T_{1\rho}$ (HS4)	96	148194		3.1 %	129	244251		0.9 %
Adiabatic $T_{1\rho}$ (HS8)	125	149642		0.4 %	150	244648		0.6 %
T_2	12	170666		9.3 %	71	197292		1.2 %
T_2*	10	638827		6.5 %	55	660116		0.5 %
Adiabatic $T_{2\rho}$	20	137472		4.5 %	82	224867		1.0 %
RAFF2	21	146686	939	5.6 %	94	240798	10598	3.6 %
CW-$T_{1\rho}$ (250 Hz)	21	132458	5456	1.1 %	81	225565	3674	0.6 %
CW-$T_{1\rho}$ (500 Hz)	41	131778	6711	2.4 %	86	223764	3393	0.3 %
CW-$T_{1\rho}$ (1000 Hz)	68	132768	6413	0.7 %	91	220387	6426	0.4 %
CW-$T_{1\rho}$ (2000 Hz)	91	134175	5113	0.6 %	100	218855	7815	0.2 %

†BL and S_{SS} refer to signal baseline in CW-$T_{1\rho}$ and the steady state signal in RAFF2.
